# Quality of life in patients with breast cancer before and after diagnosis: an eighteen months follow-up study

**DOI:** 10.1186/1471-2407-8-330

**Published:** 2008-11-11

**Authors:** Ali Montazeri, Mariam Vahdaninia, Iraj Harirchi, Mandana Ebrahimi, Fatemeh Khaleghi, Soghra Jarvandi

**Affiliations:** 1 Iranian Institute for Health Sciences Research (IHSR), ACECR, Tehran, Iran; 2 Iranian Centre for Breast Cancer (ICBC), ACECR, Tehran, Iran

## Abstract

**Background:**

Measuring quality of life in breast cancer patients is of importance in assessing treatment outcomes. This study examined the impact of breast cancer diagnosis and its treatment on quality of life of women with breast cancer.

**Methods:**

This was a prospective study of quality of life in breast cancer patients. Quality of life was measured using the European Organization for Research and Treatment of Cancer Quality of Life Questionnaire (EORTC QLQ-C30) and its breast cancer supplementary measure (QLQ-BR23) at three points in time: baseline (pre diagnosis), three months after initial treatment and one year after completion of treatment (in all 18 months follow-up). At baseline the questionnaires were administered to all suspected identified patients while both patients and the interviewer were blind to the final diagnosis. Socio-demographic and clinical data included: age, education, marital status, disease stage and initial treatment. Repeated measure analysis was performed to compare quality of life differences over the time.

**Results:**

In all, 167 patients diagnosed with breast cancer. The mean age of breast cancer patients was 47.2 (SD = 13.5) years and the vast majority (82.6%) underwent mastectomy. At eighteen months follow-up data for 99 patients were available for analysis. The results showed there were significant differences in patients' functioning and global quality of life at three points in time (P < 0.001). Although there were deteriorations in patients' scores for body image and sexual functioning, there were significant improvements for breast symptoms, systematic therapy side effects and patients' future perspective (P < 0.05).

**Conclusion:**

The findings suggest that overall breast cancer patients perceived benefit from their cancer treatment in long-term. However, patients reported problems with global quality of life, pain, arm symptoms and body image even after 18 months following their treatments. In addition, most of the functional scores did not improve.

## Background

Measuring quality of life in breast cancer patients has been the focus of clinical practice and research in recent decades and is of importance in assessing treatment outcomes [1,2]. This is partly due to the increasing number of breast cancer patients. Statistics show that each year there is over 1.1 million newly diagnosed women with breast cancer worldwide and 410,000 women die from the disease [3]. On the other hand improvement in early detection and treatment of breast cancer has led to longer survival of these patients. Also breast cancer affects women's identities and therefore studying quality of life in women who lose their breasts is vital. In addition, it is believed that women play an important role in family. When a woman develops breast cancer all family members may develop some sort of illnesses [4]. Thus the issue of 'survivorship' now has become an important topic in breast cancer care that demands the investigation of long-term effects of breast cancer diagnosis and its treatments [5].

The time of diagnosis, initial stages of adjuvant treatment course and the months immediately following the end of adjuvant treatment are transition times of poor adjustment and decreased quality of life in breast cancer patients [6,7]. Studies have shown that decreased health-related quality of life as a result of chemotherapy side effects may predict early treatment discontinuation in patients with breast cancer [8]. On the other hand studies on post-treatment adjustment of breast cancer survivors demonstrated that breast cancer patients might enjoy from a good quality of life [9].

This study empirically investigated the impact of breast cancer diagnosis and its treatments on quality of life of patients with breast cancer in an eighteen months follow-up assessment. It was thought measuring quality of life at baseline (before the final diagnosis of breast cancer was made) would provide an appropriate basis for investigating of quality of life changes over time.

## Methods

### Design

This was a prospective study of quality of life in breast cancer patients. The study was conducted in Imam Khomeini hospital during a one complete calendar year. Imam Khomeini hospital is the biggest teaching hospital in Tehran, affiliated to Tehran University of Medical Sciences. Annually about 600 to 700 both newly diagnosed and follow-up breast cancer patients are treated in Imam hospital. Imam hospital is a referral centre for most cancer patients from Tehran and other provinces. Medical consultants identified newly suspected breast cancer patients from May 2002 to May 2003. All patients were interviewed at this baseline stage. At baseline both patients and the interviewer were blind to the final diagnosis. It was assumed that being blind to the final diagnosis would provide more precise information on quality of life data. Women with confirmed histological diagnosis of breast cancer were further followed-up. There were no restrictions on patient selection with regard to histology of breast cancer, disease stage and demographic characteristics. First follow-up was scheduled three months after initial treatment and the second assessment was made one year following completion of the treatment course (18 months after pre-diagnosis stage). The course of second follow-up was completed on December 2004. Patients with progression and the end-stage cases were not followed-up. Socio-demographic data included age, education and marital status. Clinical data consisting of disease stage and initial management were extracted from case records. The Iranian Center for Breast Cancer approved the study and all interviews were carried out with patients' permissions.

### Measures

Quality of life was measured using the EORTC QLQ-C30 and its supplementary breast cancer questionnaire (QLQ-BR23). The EORTC QLQ-C30 is a well-known instrument for measuring quality of life in cancer patients and contains 30 items that measures five functional scales, global quality of life and several cancer related symptoms. The QLQ-BR23 is a specific questionnaire containing 23 items measuring functioning and symptoms related to breast cancer. The questionnaires were administered at three points in time: baseline, after initial treatment and one year after completion of treatment. The psychometric properties of the Iranian version of both questionnaires are well documented [10,11].

### Analysis

Data were analyzed using SPSS-13 and restricted to the patients that their quality of life data were available. Normality of data were examined and although distributions were slightly skewed but all skewness values were less than one allowing us to perform repeated measure ANOVA to examine changes in quality of life measures across three points in time. The P value of equal or less than 0.01 was considered significant. For comparing categorical data, chi-squared was used.

## Results

### Patients' characteristics

In all, 316 suspected breast cancer patients were interviewed at baseline (pre-diagnosis stage). Of these, 167 patients with breast cancer were followed-up. The mean age of breast cancer patients was 47.2 (SD = 13.5) years; most were married (69.4%), and have had primary or secondary education (66.5%). According to case records the vast majority of the cases (82.6%) underwent mastectomy and the disease stage was as follows: 17.4% local, 45.5% loco-regional and 37.1% metastasis. The demographic and clinical characteristics of the sample at three points in time are shown in Table 1. At first follow-up 150 breast cancer patients were re-interviewed and the remaining 17 patients were excluded from the study. Of these 17 patients, 6 patients were refused to be re-interviewed, 1 was terminally ill, 8 were lost and 2 were dead.

**Table 1:** Demographic and clinical characteristics of the study sample at three time points

	**Baseline assessment (n = 167)**	**3 months follow-up (n = 150)**	**18 months follow-up (n = 99)**	P*
	**No (%)**	**No (%)**	**No (%)**	

**Age groups (years)**				0.03
24–34	33 (19.8)	29 (19.3)	18 (18.2)	
35–44	46 (27.5)	40 (26.7)	29 (29.3)	
45–54	39 (23.4)	38 (25.3)	30 (30.2)	
55–64	28 (16.8)	26 (17.3)	15 (15.2)	
64 >	21 (12.5)	17 (11.4)	7 (7.1)	
Mean (SD)	47.2 (13.5)	47.4 (13.3)	46.4 (12.5)	
Range	24–81	24–81	24–81	
**Educational level**				0.05
Illiterate	38 (22.8)	34 (22.7)	17 (17.2)	
Primary	78 (46.7)	71 (47.3)	45 (45.5)	
Secondary	33 (19.8)	29 (19.3)	25 (25.2)	
College/university	18 (10.7)	16 (10.7)	12 (12.1)	
**Marital status**				0.4
Single	15 (9.0)	13 (8.7)	11 (11.1)	
Married	116 (69.4)	105 (70.0)	67 (67.7)	
Widowed	36 (21.6)	32 (21.3)	21 (21.2)	
**Disease stage**				0.4
Local	29 (17.4)	25 (16.7)	18 (18.2)	
Loco-regional	76 (45.5)	71 (47.3)	48 (48.5)	
Metastasis	62 (37.1)	54 (36.0)	33 (33.3)	
**Initial management**				0.09
Mastectomy	138 (82.6)	125 (83.3)	84 (84.8)	
Conservative surgery	15 (9.0)	14 (9.3)	11 (11.1)	
Chemotherapy	11 (6.6)	10 (6.7)	4 (4.1)	
Best supportive care	3 (1.8)	1 (0.7)	0 (0.0)	

*χ^2 ^test

At the second follow-up 99 patients were interviewed and the remaining 51 patients were excluded from the study. Reasons for attrition were: dislike (n = 25), loss to follow (n = 10) and death (n = 16). Analysis showed that women who completed the entire study differed on age and educational level from the sample that dropped out of the study.

### Functioning and global quality of life

Table 2 shows patients' functioning and global quality of life as measured by the EORTC QLQ-C30. Physical functioning improved over time while there was deterioration in most of the other functioning scales.

**Table 2:** Breast cancer patients' pre-diagnosis and follow-ups functioning and global quality of life scores as measured by the EORTC QLQ-C30* (n = 99)

	**Baseline assessment**	**3 months follow-up**	**18 months follow-up**	**P**
**Functioning**	**Mean (SD)**	**Mean (SD)**	**Mean (SD)**	

Physical functioning	68.7 (24.9)	66.8 (20.6)	72.6 (19.7)	<0.001
Role functioning	69.7 (27.1)	66.0 (24.5)	69.8 (30.4)	<0.001
Emotional functioning	59.4 (23.5)	61.3 (24.1)	55.5 (27.6)	<0.001
Cognitive functioning	79.4 (20.1)	74.2 (19.2)	74.4 (20.6)	<0.001
Social functioning	85.0 (18.0)	82.5 (20.9)	79.3 (25.5)	<0.001
Global quality of life	59.2 (31.8)	71.3 (25.6)	32.0 (30.2)	<0.001

*The higher values indicate higher level of functioning and quality of life, min: 0, max: 100

Physical functioning was increased by 5.8 point after 18 months follow-up, which could be regarded remarkable improvement. Global quality of life scores showed a fluctuated picture: 59.2 at baseline, 71.3 at 3-months follow-up and 32.0 at third assessment.

### Symptoms

The analysis showed that in all measures there were statistically significant differences in patients' symptom scores (Table 3). Compared to the baseline and three months assessments, symptoms including fatigue, pain and dyspnea were increased at 18 months follow-up assessment.

**Table 3:** Breast cancer patients' pre-diagnosis and follow-ups symptoms scores as measured by the EORTC QLQ-C30* (n = 99)

	**Baseline assessment**	**3 months follow-up**	**18 months follow-up**	**P**
**Symptoms**	**Mean (SD)**	**Mean (SD)**	**Mean (SD)**	

Fatigue	17.0 (19.4)	31.0 (25.4)	36.0 (21.8)	<0.001
Nausea and vomiting	1.7 (7.3)	29.3 (30.5)	2.5 (10.7)	<0.001
Pain	4.5 (9.7)	4.5 (10.6)	15.3 (21.0)	<0.001
Dyspnea	5.7 (14.3)	8.7 (16.2)	14.8 (21.9)	<0.001
Sleep difficulties	27.5 (30.2)	25.2 (30.3)	26.9 (27.3)	<0.001
Appetite loss	20.9 (29.2)	35.7 (32.4)	6.7 (18.4)	<0.001
Constipation	2.3 (9.8)	9.1 (19.5)	10.1 (19.9)	<0.001
Diarrhea	0.33 (3.3)	2.0 (9.3)	1.0 (5.7)	0.003
Financial difficulties	17.7 (25.8)	22.1 (27.0)	23.1 (28.5)	<0.001

*The higher values indicate a greater degree of symptoms, min: 0, max: 100

Fatigue as the most disturbing disease- and treatment-related symptom was increased sharply both at 3-months assessment and 18-months follow-up: 17.0 at baseline, 31.0 at 3-months follow-up and 36.0 at 18 months follow-up. Also at 3 and 18 months follow-ups there were higher levels of financial difficulties than baseline (22.1 and 23.1 versus 17.7).

### Breast cancer specific scores

Breast cancer patients' quality of life scores as measured by the EORTC QLQ-BR23 are shown in Table 4. Except for future perspective, there were significant deteriorations in all other patients' functioning scores over time compared to the baseline assessment.

**Table 4:** Breast cancer patients' pre-diagnosis and follow-ups functioning and symptoms scores as measured by the EORTC QLQ-BR23* (n = 99)

	**Baseline assessment**	**3 months follow-up**	**18 months follow-up**	**P**
	**Mean (SD)**	**Mean (SD)**	**Mean (SD)**	

**Functioning**				
Body image	86.2 (18.8)	61.4 (34.4)	60.8 (34.4)	<0.001
Sexual functioning	82.3 (22.4)	74.5 (21.9)	67.7 (26.4)	<0.001
Sexual enjoyment	51.6 (25.3)	46.6 (25.1)	23.3 (28.8)	<0.001
Future perspective	29.9 (29.5)	36.0 (26.4)	36.7 (29.1)	<0.001
**Symptoms+**				
Arm symptoms	7.7 (13.3)	17.2 (16.5)	24.8 (20.2)	<0.001
Breast symptoms	15.6 (15.0)	10.2 (14.0)	6.5 (9.7)	<0.001
Systematic therapy side effects	15.7 (11.8)	29.2 (17.5)	18.0 (13.7)	<0.001
Upset by hair loss	0.00	38.9 (38.9)	50.0 (18.2)	0.003

* Scores range from 0 to 100, with higher scores representing a higher level of functioning.

+ Scores range from 0 to 100, with higher scores representing a higher level of symptoms.

Decreased body image was observed at 3 and 18 months follow-ups (61.4 and 60.8 respectively) compared to the baseline assessment (86.2).

Comparing the baseline assessment, an increased level of arm symptoms was observed at 3 months follow-up (17.2) that continued to be persistent at 18 months follow-up (24.8).

## Discussion

This study provided data on health-related quality of life of 99 breast cancer patients through an eighteen months follow-up using standard quality of life measures. The results showed that physical functioning was improved following one year after the completion of breast cancer treatment (Table 2). It is argued that most aspects of health-related quality of life including physical health will recover after adjuvant treatment course ends and no residual effects will exist in longer periods for the majority of patients [12-14].

Patients rated their emotional functioning lower at 18 months follow-up than baseline and 3 months assessments. Similarly, the patients at 18 months follow-up reported decreased global quality of life. A study on distress and quality of life 3 months following treatment for breast cancer patients showed that there were moderate distress due to fear of cancer recurrence and resuming normal life. However, the same study found that quality of life, as measured by the SF-36 questionnaire, was improved in most areas including physical functioning, bodily pain and vitality [9]. It is believed that 1 to 3 months following adjuvant treatment, as a transition period, is a time of disruption and increased distress [7,15]. There might be several explanations why this post-treatment period is a particularly distressing time for breast cancer patients. Remaining the physical effects of treatment such as fatigue, hair loss, lymph edema [16], leaving their routine connection with their medical treatment team [7] and loss of support from family and friends who may not realize patients' cancer-related physical and psychological issues [16] are among the most important reasons. A qualitative study among breast cancer patients who had completed their treatment investigated their health care needs. The findings indicated that these women continue to experience a variety of physical and psychological symptoms and need information and support [17].

In this study patients reported poor social functioning following completion of breast cancer treatment. Similarly studies have found that breast cancer survivors suffer from poor social functioning [18,19].

Decreased cognitive functioning was observed at 3 months assessment that continued to be persistent at 18 months follow-up. Long-term (1 to 10 years) cognitive impairment in patients with breast cancer after their chemotherapy treatment has been reported [20,21]. It is argued that the observed impairment occurs most often in attention, learning and processing speed and is not attributed to demographic characteristics, clinical features and baseline level of cognitive function [20].

There were elevated levels of fatigue, pain and dyspnea (Table 3) and arm symptoms (Table 4) at 18 months follow-up assessment. This is consistent with the findings of similar studies that reported women with breast cancer experienced substantial complaints as a result of cancer and its treatment [22-24]. A review on adjuvant systemic therapy for early stage breast cancer reported that except for vasomotor symptoms all the other detrimental effects of these treatments are transient and would rapidly be removed at the end of the treatment courses [2].

Except for future perspective all the other breast cancer specific functioning including body image, sexual functioning and sexual enjoyment decreased at eighteen months follow-up assessment. Sexual dysfunction is a symptom that may occur as a result of premature menopause following adjuvant systemic therapy in breast cancer patients [2].

Most patients in this study were diagnosed with advanced disease (loco-regional 45.5%, and metastasis 37.1%). Also mastectomy was the treatment of choice for 82.6% of patients and just a small number of them received breast conservation (9.0%). A study on cancer practice by general surgeons in Iran showed that Iranian surgeons do not routinely perform breast conserving surgery as the first treatment modality for breast cancer patients [25]. Unfortunately this is a common situation in Iran and it needs further attention in order to improve both early diagnosis and clinical outcomes.

However this study was limited due to its small cohort of breast cancer patients. Also there was a drop-out-rate of nearly one third of patients during the follow-up courses. In addition, patients were relatively young, although studies have shown that in Iran breast cancer patients present with advanced stage and they are about 10 years younger than their western counterparts [26,27]. Furthermore most of the functional scores did not improve over time and this is in contradiction to the findings from some existing literature [28].

## Conclusion

The study findings showed that overall breast cancer patients perceived benefit from their adjuvant treatment. However sustained problems such as fatigue, pain, sleep disturbances and arm symptoms were observed. Indeed, these should be managed by targeted interventional programs. Also, impaired body image decreased sexual functioning and sexual enjoyment in patients must be seriously considered in long-term survivors of breast cancer to improve their overall quality of life.

## Abbreviations

EORTC QLQ-C30: European Organization for Research and Treatment of Cancer Quality of Life Questionnaire-Cancer 30; EORTC QLQ-BR23: European Organization for Research and Treatment of Cancer Quality of Life Breast Cancer Questionnaire.

## Competing interests

The authors declare that they have no competing interests.

## Authors' contributions

AM was the main investigator, analyzed the data, and wrote the paper. MV collected the data, contributed to the analysis and helped in writing the final manuscript. IH helped in study design and patient recruitment. ME, FK and SJ contributed to the study design and the data collection. All authors read and approved the final manuscript.

## Pre-publication history

The pre-publication history for this paper can be accessed here:


